# A high-risk luminal A dominant breast cancer subtype with increased mobility

**DOI:** 10.1007/s10549-019-05135-w

**Published:** 2019-02-19

**Authors:** Liping Guo, Guoji Chen, Wen Zhang, Lulin Zhou, Ting Xiao, Xuebing Di, Yipeng Wang, Lin Feng, Kaitai Zhang

**Affiliations:** 1 State Key Laboratory of Molecular Oncology, Department of Etiology and Carcinogenesis, National Cancer Center/National Clinical Research Center for Cancer/Cancer Hospital, Chinese Academy of Medical Sciences and Peking Union Medical College, Beijing, 100021 China; 2 Department of Breast Surgery, National Cancer Center/National Clinical Research Center for Cancer/Cancer Hospital, Chinese Academy of Medical Sciences and Peking Union Medical College, Beijing, 100021 China; 3 Department of Immunology, National Cancer Center/National Clinical Research Center for Cancer/Cancer Hospital, Chinese Academy of Medical Sciences and Peking Union Medical College, Beijing, 100021 China

**Keywords:** Breast cancer classification, t-SNE, Biology process, Immune pattern, NETs

## Abstract

**Purpose:**

Breast cancer is a heterogeneous disease, and although advances in molecular subtyping have been achieved in recent years, most subtyping strategies target individual genes independent of one another and primarily concentrate on proliferative markers. The contributions of biological processes and immune patterns have been neglected in breast cancer subtype stratification.

**Methods:**

We performed a gene set variation analysis to simplify the information on biological processes using hallmark terms and to decompose immune cell data using the immune cell gene terms on 985 breast invasive ductal/lobular carcinoma RNAseq samples in the TCGA database.

**Results:**

The samples were gathered into three clusters following implementation of the t-SNE and DBSCAN algorithms and were categorized as ‘hallmark-tsne’ subtypes. Here, we identified a high-risk luminal A dominant breast cancer subtype (C3) that displayed increased motility, cancer stem cell-like features, a higher expression of hormone/luminal-related genes, a lower expression of proliferation-related genes and immune dysfunction. With regard to immune dysfunction, we observed that the motility-increased C3 subtype exhibited high granulocyte colony stimulating factor (G-CSF) expression accompanied by neutrophil aggregation. Cancer cells that produce high levels of G-CSF can stimulate neutrophils to form neutrophil extracellular traps, which promote cancer cell migration. This finding sheds light on one potential explanation for why the C3 subtype correlates with poor prognosis.

**Conclusions:**

The hallmark-tsne subtypes confirmed again that even the luminal A subtype is heterogeneous and can be further subdivided. The biological processes and immune heterogeneity of breast cancer must be understood to facilitate the improvement of clinical treatments.

**Electronic supplementary material:**

The online version of this article (10.1007/s10549-019-05135-w) contains supplementary material, which is available to authorized users.

## Introduction

Breast cancer is commonly considered a collection of heterogeneous diseases with completely different treatment schemes and clinical outcomes rather than a consistently defined single disease affecting the same organ [1]. Hence, diverse classification methods have emerged to predict the prognosis or assist with clinical treatment decisions, particularly methods based on gene expression signatures, such as the ‘intrinsic’ genes [2], the PAM50 signature [3] in classical molecular subtypes, and the MammaPrint signature [4], the Wang76 genes [5] and OncotypeDX [6] for predicting prognosis. However, the signatures selected in the last few decades to act as prognostic and predictive factors have concentrated on proliferative markers [7]. Other biological processes influencing tumorigenesis and prognosis have been neglected by traditional analyses. In addition, although the estrogen receptor (ER), progesterone receptor (PR) and epidermal growth factor receptor 2 (HER2) status are classical molecular subtypes that are prevailingly applied in clinical practice, the categorization is not sufficient to distinguish certain minor subtypes and therefore fails to assist with all treatment decisions [8].

Unlike traditional tumor molecular signature mining, we hypothesized that biological processes and the tumor-infiltrating immune pattern might be related to prognosis and subtype stratification. Specifically, the gene terms associated with certain biological processes or immune cells rather than one single gene are more powerful for evaluating the intrinsic nature of a given cancer. The hallmarks, which are a refined gene set, are derived from the original gene sets in the Molecular Signatures Database (MSigDB), and these convey a specific biological state or process and display coherent expression [9]. Furthermore, the deconvolution of gene expression profiles of infiltrating immune cells from those of bulk tumors is now possible [10] through CIBERSORT [11] and DeconRNA-Seq [12] methods. To computationally and coordinately evaluate the biological processes and immune cell patterns, we simplified the biological process information using hallmark terms and decomposes the immune cell using immune cell gene terms through gene set variation analysis (GSVA) [10], which is a gene set enrichment analysis, according to Senbabaoglu et al. [13].

Interestingly, we discovered a high-motility high-risk luminal A dominant breast cancer type (referred to as C3 hereafter) in which the phenotype and clinical outcome are completely different from the traditional luminal subtype. We observed that the motility-increased C3 subtype expressed high levels of granulocyte colony stimulating factor (G-CSF) and showed neutrophil aggregation, consistent with the phenomenon that certain cancer cells can stimulate neutrophils to form neutrophil extracellular traps (NETs) and thereby support cancer cell migration and invasion [14]. Thus, characterizing the minor C3 subtype has pressing clinical implications with regard to specific treatments, such as deoxyribonuclease I (DNAase I) to digest NETs or the use of a targeted antibody to neutralize IL-8 and decrease neutrophil recruitment [15].

## Materials and methods

### Datasets

The dataset, including mRNA expression ‘level3’ data (RNAseqV2, RSEM) and the clinical characteristics of breast invasive ductal/lobular carcinoma (IDC/ILC), was downloaded from the TCGA database. IDC/ILC samples (*n* = 985) were classified by the ‘intrinsic gene subtype’ (luminal A, luminal B, HER2-enriched, basal-like and normal-like) [2, 16]. The mRNA expression matrix was transformed by log2(*x* + 1).

The 1868 IDC/ILC samples from Molecular Taxonomy of Breast Cancer International Consortium (METABRIC) datasets with intact clinical information (categorized as ‘Claudin-low’ [17] and ‘intrinsic gene subtype’) were retrieved from cBioPortal [18].

### Gene sets

The hallmark gene sets [9], including 4386 genes in 50 terms, were downloaded from the Molecular Signatures Database v6.0 (MSigDB). The second gene sets included the gene signatures used for the decomposition of immune cell types, angiogenesis marker genes and signatures of antigen presentation, as described by Senbabaoglu et al. [13]. The third gene set, which consisted of exhausted T cells, was defined by two criteria (Table S1) [19].

### Hallmark-GSVA enrichment scores (HGSs) and hazards analysis

GSVA is a nonparametric, unsupervised method that can condense information from gene expression profiles into a pathway or a signature summary [10]. Using the R package ‘GSVA’, each sample received 50 scores corresponding to 50 hallmark gene terms, and the enrichment scores are hereafter referred to as HGSs.

To evaluate the prognostic ability of the hallmark terms, we performed a univariate Cox proportion hazards regression analysis using the R package ‘survival’. To remove redundancies, a correlation analysis (Pearson’s correlation analysis) was performed with the HGSs of the remaining prognosis-associated terms.

### Unsupervised clustering, prognostic differentially expressed genes (P-DEGs) identification and survival analysis among clusters

With the expression matrix of 2136 genes from the prognosis-associated terms, the Euclidean distance was calculated between any two samples and condensed into two-dimensional points using a nonlinear dimensionality reduction algorithm (t-distributed stochastic neighbor embedding (t-SNE)) [20] and subsequently visualized automatically with the density-based spatial clustering of applications with noise (DBSCAN) algorithm. The above processes were performed using the R packages ‘Rtsne’ and ‘dbscan’.

**Fig. 1:**
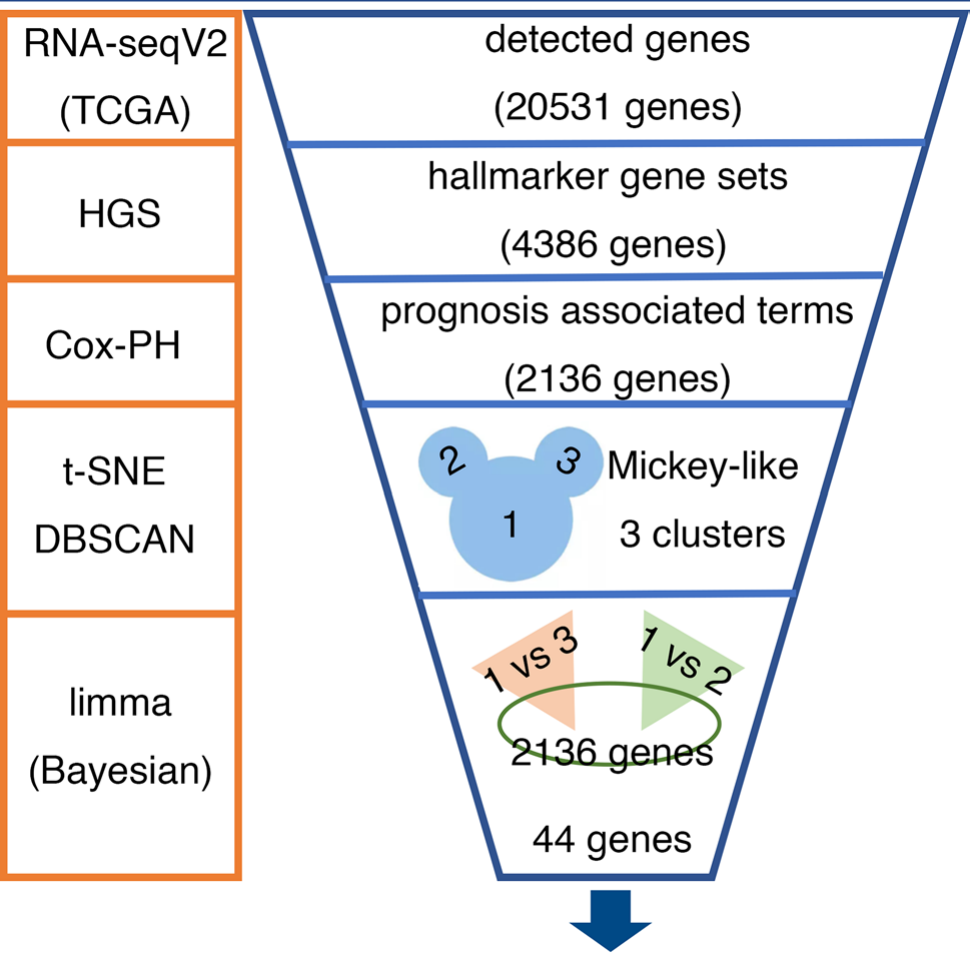
Pipeline for identifying the 44 predominant P-DEGs. RNA-seqV2 (TCGA): mRNA expression datasets from The Cancer Genome Atlas (TCGA). HGS: The hallmark-GSVA enrichment score was calculated using hallmark gene sets with the gene set variation analysis algorithm. Cox-PH: Cox’s proportional hazards regression model. t-SNE: t-distributed stochastic neighbor embedding. DBSCAN: density-based spatial clustering of applications with noise. limma (Bayesian): the R package ‘limma’ was used with the empirical Bayesian model to identify the differentially expressed genes

Linear models and empirical Bayes methods were applied to distinguish the differentially expressed genes (DEG) among clusters using the R package ‘limma’. The top 44 significantly different genes were selected from the 2136 genes and were defined as dominant prognostic differentially expressed genes (P-DEGs) for the following analysis. The 44 P-DEGs were divided into clusters based on a hierarchical cluster analysis according to their expression counts. The pipeline used to identify the 44 predominant P-DEGs is shown in Fig. 1.

Survival curves were calculated using the Kaplan–Meier method, with the log-rank test (two-tailed) for hypothesis testing, and the Cox model was performed using the R packages ‘survival’ and ‘ggplot2’.

### Decomposition of the tumor-infiltrating cells among the clusters

Similar to the HGSs, the GSVA algorithm was used to quantify the composition of the 24 immune cells and their subtypes, the levels of angiogenesis, the conditions of antigen presentation and the potential of exhausted T cells.

### Breast cancer cell lines

The MCF-7 cell line, which was provided by Professor Ningzhi Xu, and the MDA-MB-231 cell line, which was maintained in our labs, were cultivated in RPMI-1640 medium (HyClone Laboratories; SH30809.01B) supplemented with 10% fetal bovine serum (FBS; Gibco; 10099-141) and 100 units/mL penicillin and streptomycin.

### Quantitative reverse transcription PCR (qRT-PCR)

Total RNA was isolated using TRIzol reagent (Invitrogen; 15596018) and reversed transcribed with Superscript II (Invitrogen; 18064014) according to the manufacturer’s instructions. The SYBR Green method was used to detect the expression of colony stimulating factor 3 (CSF3, also called G-CSF) along with the endogenous control GAPDH. The primers for CSF3 and GAPDH were as follows: CSF3, forward, 5′-GAAGCTGGTGAGTGAGTGTGC-3′ and reverse, 5′-GGTAGAGGAAAAGGCCGCTA-3′; and GAPDH, forward, 5′-ACAACTTTGGTATCGTGGAAGG-3′ and reverse, 5′-GCCATCACGCCACAGTTTC-3′. The assay was performed in triplicate for each sample.

## ELISA

The undiluted cancer cell culture supernatants and empty culture medium (control) were assayed for G-CSF using the Human G-CSF Immunoassay Kit (R&D Systems; DCS50).

### Transwell migration assay

Peripheral venous blood was collected into K2EDTA vacuum tubes (BD Biosciences; 367844) from volunteers. Polymorphonuclear cells (PMNs), mainly neutrophils, were separated immediately after venipuncture using Polymorphprep (Axis-Shield) according to the manufacturer’s instructions. The lower cell band, neutrophil predominated, was collected, washed with PBS without Ca^2+^/Mg^2+^ (Gibco; 10010023) and resuspended in erythrocyte lysis buffer (Qiagen; 79217) to remove residual red blood cells. After centrifugation, the cell pellet was washed and resuspended in serum-free RPMI-1640 medium.

The purity of the isolated neutrophils was determined by flow cytometry using anti-CD15 PE (clone HI98; eBioscience; 12-0159-42), anti-CD16 APC (clone 3G8; Biolegend; 302012) and anti-CD49d (clone 9F10; Biolegend; 304316) according to the manufacturer’s recommendation. Meanwhile, the isolated neutrophils were confirmed by counting cells with a multilobular nucleus after staining with Hoechst 33342 (1:1000 diluted, Invitrogen; H3570).

The prepared neutrophils were plated in the lower chamber that contained poly-L-lysine-coated coverslips. After 15 min, blocking anti-G-CSF antibodies (Abcam; ab9691) or human recombinant G-CSF (6 ng/mL; Proteintech; HZ-1207) or the vehicle was added. The cancer cells were seeded in the top chamber with a pore size of 8 µm (Corning; 3422). Three hours later, 10% FBS (Gibco; 10099-141) was added to the lower chamber. After 22 h, the cells were fixed, stained with crystal violet and counted at 400x magnification in 5 fields under a microscope. The assays were repeated at least three times.

### Detecting neutrophil extracellular trap (NET) formation

Unstimulated neutrophils, neutrophils stimulated with 20 nM PMA (MCE, HY-18739), and neutrophils cocultured with cancer cells were seeded on coverslips, fixed, permeabilized and blocked. Subsequently, the cells were stained with anti-myeloperoxidase antibodies [2C7] (1:400 diluted, Abcam; ab25980), anti-histone H3 (citrulline R2 + R8 + R17) antibodies (1:400 diluted, Abcam; ab5103), Hoechst 33342 (1:1000 diluted, Invitrogen; H3570) and their corresponding secondary antibodies, goat anti-mouse IgG (Alexa 488) (1:2000 diluted, Abcam; ab150113) and goat anti-rabbit IgG (Alexa 568) (1:2000 diluted, Abcam; ab175471). The stained coverslips were visualized with a confocal laser scanning microscopy platform (Leica TCS SP8).

### Quantification of NET formation by MPO:DNA complexes

High-binding 96-well microplates (costar, 42592) were coated overnight at 4 °C with mouse anti-human MPO (1:500 diluted, AbD Serotec; 0400-0002). After blocking with 1% BSA (Sigma; A3803) for two hours at room temperature, 1:1 diluted condition cell supernatants were added and incubated for 2 h at room temperature and washed, and anti-DNA-peroxidase conjugated antibody (1:22 diluted, Roche, 11774425001) was added for 1 h at room temperature. Subsequently, TMB substrate (Abcam; ab171523) was added and evaluated at 450 nm after the addition of stop solution (Sigma, ab210900).

### Statistical analysis

The analysis was performed with R 3.4.3 and GraphPad Prism 6. The results are presented as the means ± SEMs. One-way analysis of variance (ANOVA) was used to evaluate the expression differences of the ‘neutrophils’, ‘T gamma delta cells (Tgd)’ and ‘G-CSF (CSF3)’ among the three clusters, and the Bonferroni correction was used with a pairwise comparison. T tests were used to evaluate the difference in the expression counts of CSF3 between the MCF7 and MDA-MB-231 cell lines. One-way ANOVA was also used to evaluate the different secretion counts of G-CSF among the four conditioned mediums. The transwell migration assay was analyzed by two-way ANOVA with multiple comparisons. All tests were two-sided with a significance level of 0.05.

## Results

### Prognosis-associated hallmark gene sets

**Fig. 2:**
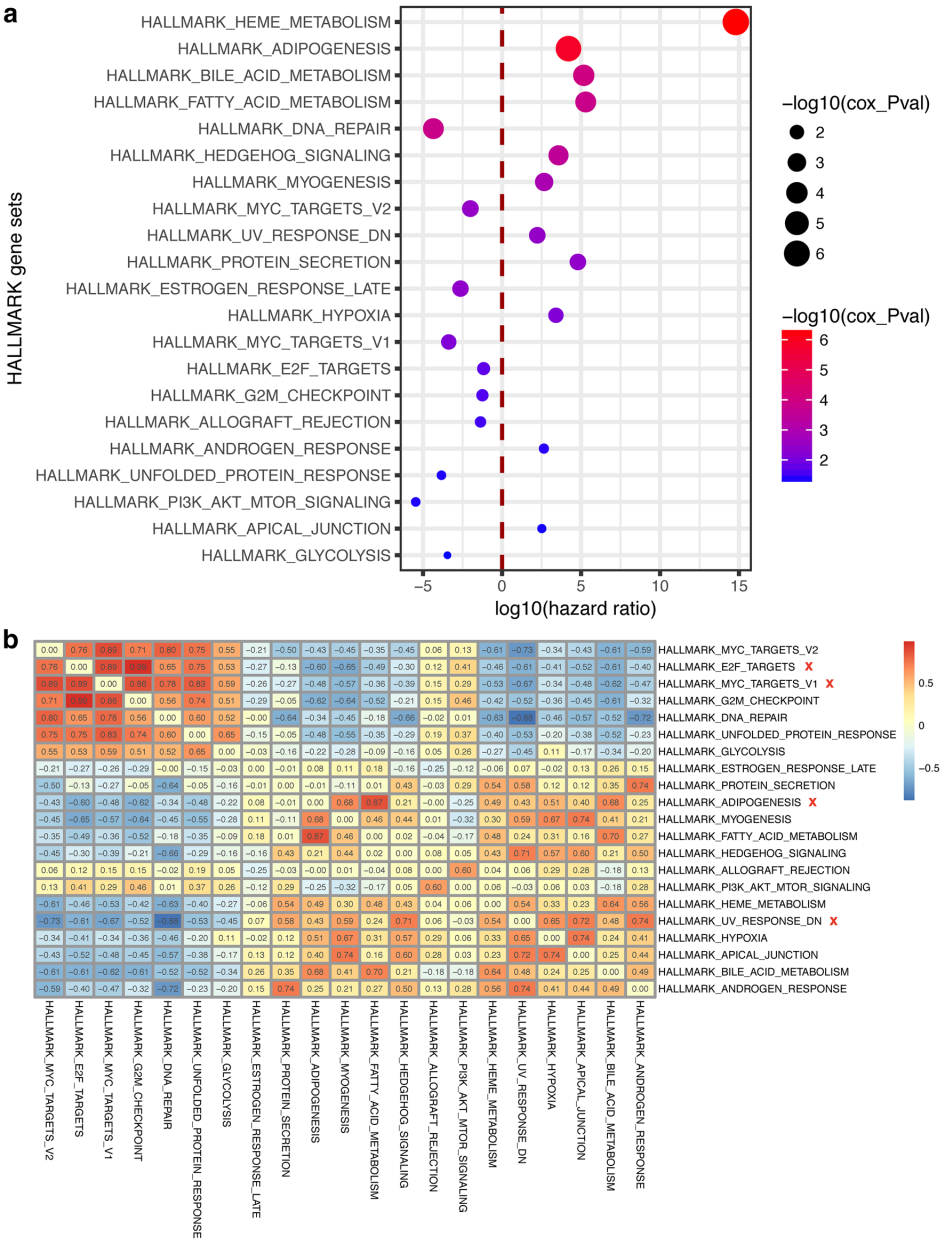
Hallmark-GSVA score (HGS) and prognosis. **a** Twenty-one prognosis-associated hallmark gene sets and their hazard ratios. The hazard ratios were logarithmically transformed; that is, the transformed ratios of less than zero correspond to protective factors, and ratios of more than zero correspond to risk factors (abscissa). The red dashed line divides the protective and risk factors. The hallmark gene sets are shown based on ascending order of *p* values. The diameter or color of the circle depicts the *p* value. **b** Correlation among each hallmark gene set. The removed hallmark terms are marked with a red ‘x’

Each breast IDC/ILC sample was given a HGS according to their mRNA expression data (see “Materials and methods” section for details). Twenty-one prognosis-associated hallmark gene sets were distinguished according to their HGS using a univariate Cox proportional hazards regression. Overall, the hazardous hallmarks were focused on two processes: the material metabolism that is involved with heme, fatty acid and bile acid; and development, including hedgehog signaling, myogenesis and adipogenesis. At odds with the conventional consensus, protective hallmarks were concentrated on not only DNA repair and immune rejection but also tumor-specific behavior, proliferative capacity and PI3K-AKT-mTOR signaling (Fig. 2a). However, it is reasonable that these aspects pertain to protective factors considering the corresponding targeted medicine, such as the CDK and mTOR inhibitors that are currently applied in breast cancer patient treatments [21, 22].

To elucidate the typical prognosis-associated biological behaviors and to simplify the core prognosis genes, a correlation analysis was performed with the HGSs of twenty-one hallmarks. Eventually, four terms were removed: HALLMARK_MYC_TARGETS_V1, HALLMARK_E2F_TARGETS and HALLMARK_ADIPOGENESIS showed highly positive correlations with HALLMARK_MYC_TARGETS_V2, HALLMARK_G2M_CHECKPOINT, and HALLMARK_FATTY_ACID_METABOLISM, respectively; and HALLMARK_UV_RESPONSE_DN was inversely related to HALLMARK_DNA_REPAIR. Finally, 17 HALLMARKs, as well as the 2136 genes, were retained (Fig. 2b).

### Mickey-like clusters

**Fig. 3:**
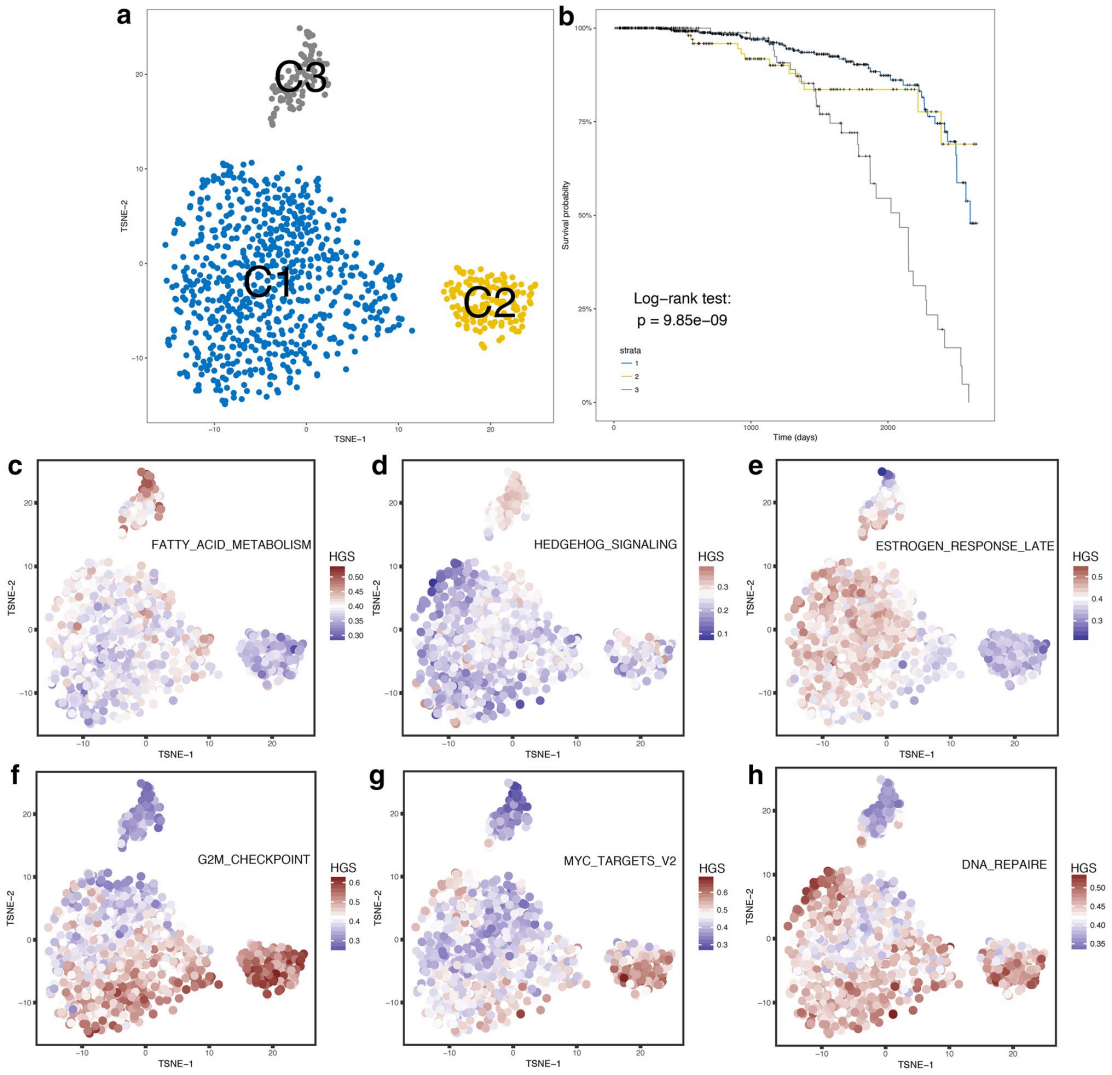
Mickey-like clusters and their HGS profiles. The distributions of the 985 IDC/ILC samples are based on 2136 genes and their survival curves in (**a**) and (**b**), respectively. Blue, yellow and gray represent the C1, C2 and C3 subtypes, respectively. The subgraphs of C-H represent the HALLMARK_FATTY_ACID_METABOLISM GSVA score profile (**c**), the HALLMARK_HEDGEHOG_SIGNALING GSVA score profile (**d**), the HALLMARK_ESTROGEN_RESPONSE_LATE GSVA score profile (**e**), the HALLMARK_G2M_CHECKPOINT GSVA score profile (**f**), the HALLMARK_MYC_TARGETS_V2 GSVA profile (**g**) and the HALLMARK_DNA_REPAIRE GSVA score profile (**h**) of the Mickey-like clusters. Higher expression counts feature higher HGSs in brownish red; lower expression counts are shown in blue. The top three graphs exhibit high HGS terms in C3 but low in C2, whereas the following three graphs exhibit high HGS terms in C2 but not in C3

The 985 IDC/ILC samples from TCGA, with the expression matrix of 2136 genes, were grouped into three clusters (Fig. 3a). Furthermore, the 44 predominant P-DEGs are representative of the fact that they still have the power to divide the sample into three clusters, the main part remaining the same as the clusters divided by 2136 genes, except for 8 redistributed samples and 4 unclassified samples (Fig. S1). The three clusters were named ‘Mickey-like’ clusters because of their special distribution shape or referred to as the ‘hallmark-tsne’ subtype for classification standard. The major part was classified as C1, and the other two small parts, the ‘Mickey’s ears’, were classified as C2 and C3 subtypes. The clustering method and the definition of the 44 P-DEGs are described in the “Materials and Methods”.

The survival analysis indicated entirely different prognoses among the three clusters (Fig. 3b and Table S2) (log-rank test, *p* < 0.0001). The C1 subtype patients tended to exhibit a longer survival than the C2 and C3 patients. The worst outcome was observed in the C3 patients. Additionally, we adjusted for the effect of clinical stage and intrinsic subtypes to assess the independent prognostic factors with a multivariate Cox proportional hazards model. The results indicated that the hallmark-tsne type is an independent prognostic factor (C3 hazard ratio: 4.84; 95% confidence interval: 2.96–7.93; *p* = 3.74 × 10^−10^); details can be found in Table 1.

**Table 1:** Adjusted results of the Cox proportional hazards regression model

	HR	CI 2.50%	CI 97.50%	*p*
Hallmark-tsne subtype				
C1	1			
C2	1.122978794	0.513001607	2.458240588	0.77169682
C3	4.839769338	2.955063741	7.926518443	3.74 × 10^− 10^
Intrinsic subtype				
BasL	1			
ErbB2	1.134277317	0.392047279	3.281708869	0.816188878
LumA	0.367733918	0.16587466	0.815243479	0.013783873
LumB	0.551906583	0.219497433	1.387719541	0.20642468
NormL	0.982353851	0.123123789	7.837795537	0.986594242
notSure	0.437117477	0.193527858	0.987308448	0.046515083
Stage	2.268524628	1.672357399	3.077215427	1.40 × 10^− 07^

*HR* Hazard ratio, *CI* confidence interval, *C2* cluster 2 based on the hallmark-tsne subtypes, *C3* cluster 3 based on the hallmark-tsne subtypes, *BasL* basal like, *ErbB2* ErbB2/HER2-enriched, *LumA* luminal A, *LumB* luminal B, *NormL* normal-like, *notSure* types are uncertain

From the HGS profile of the Mickey-like clusters, we hypothesized that the different distributions of HGSs among the three clusters were correlated with their diverse prognostic outcomes. The HGS profile of the Mickey-like clusters is shown in Fig. 3c–h. These results indicated that the worst C3 cancers exhibited active fatty acid metabolism, stemness features (high expression of hedgehog signaling) and luminal cancer characteristics (high expression of estrogen-related genes). Conversely, the C2 subtype results focused on the cell proliferation phenotype. Surprisingly, the worst C3 cancers did not reveal a high proliferative ability.

### High-risk subgroup in luminal A breast cancer

**Fig. 4:**
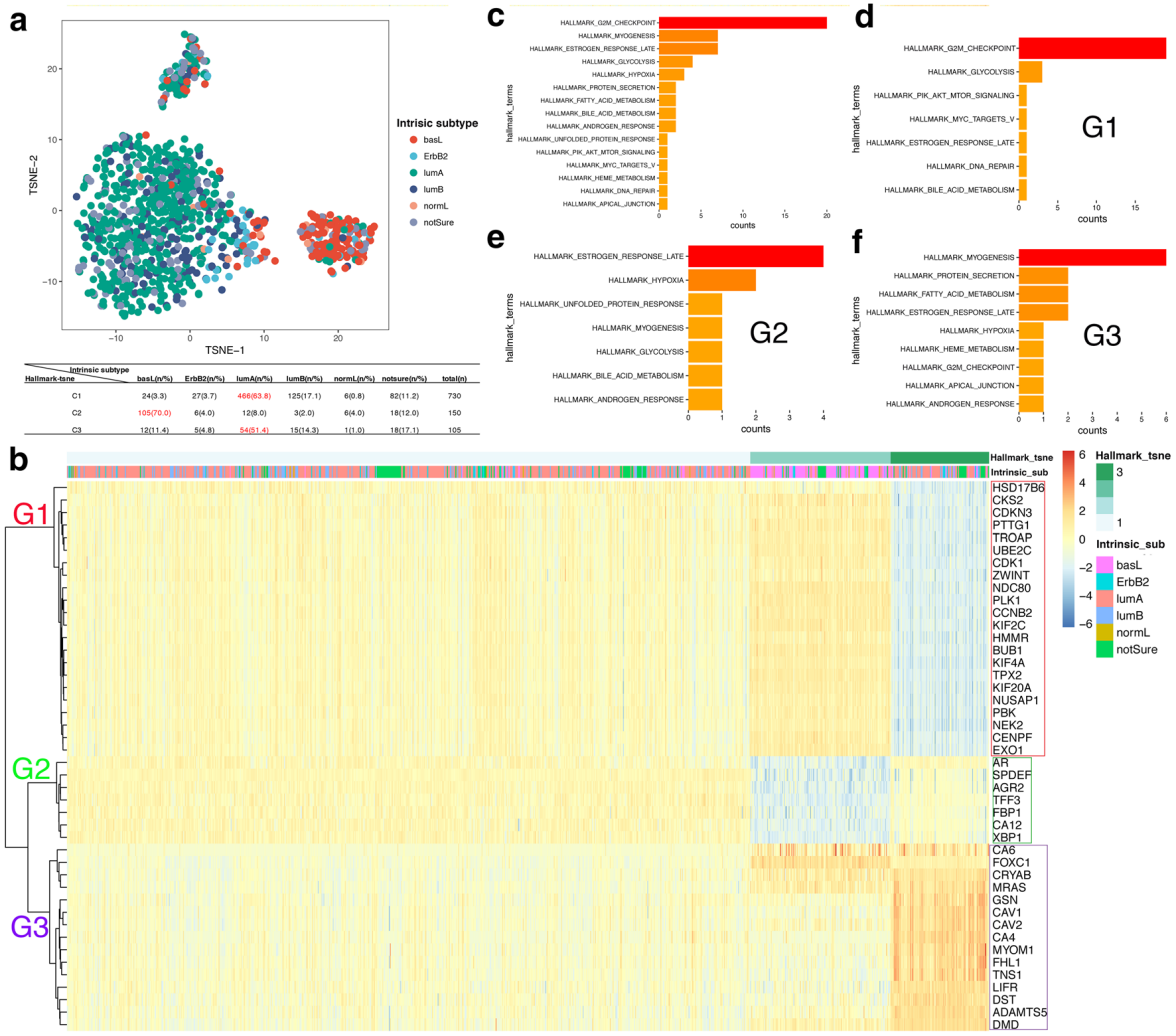
Characteristics of the Mickey-like clusters. **a** The distribution of the intrinsic gene subtype in the hallmark-tsne subtype. The frequency and percentage of intrinsic subtypes in hallmark-tsne types are shown in the table **a**; the dominant subtypes in each hallmark-tsne type are marked in red. **b** Clustered heatmap of 44 P-DEGs in the Mickey-like clusters. The heatmap shows the expression count of the 44 predominant P-DEGs among the three Mickey-like clusters. The genes in G1, G2 and G3 were labeled with red, green and purple rectangles, respectively. **c**–**f** Divided three groups of P-DEGs and their corresponding HALLMARK terms. All 44 genes and their corresponding HALLMARK terms are shown in (**c**). The G1, G2 and G3 genes are exhibited in (**d**), (**e**) and (**f**), respectively

To some extent, the patients, based on their intrinsic gene subtypes, scattered themselves into the hallmark-tsne subtypes. Specifically, each Mickey-like cluster was blended with all types of intrinsic gene subtypes but retained one dominant type. For example, the luminal A subtype was particularly represented and comprised a large part of the C1 and C3 subtypes; however, a large proportion of C2 consisted of the basal-like subtype (Fig. 4a; Table 2). Moreover, the samples were classified as C1 and C3, which were ER and PR positive, in contrast to the C2 samples (Fig. S2a and b). Similarly, HER2 positive samples were mainly in C1 and C3 subtypes (Fig. S2c). Interestingly, the luminal A patients separated into two clusters (C1 and C3) that displayed completely different prognoses (Fig. S3). Therefore, there is an extremely high-risk subgroup of luminal A cancer. Similar results showing that luminal A cancer is a collection of heterogeneous diseases have been reported based on a copy number alteration analysis [23].

**Table 2:** Distribution of classical molecular subtypes and hallmark-tsne subtypes in breast cancer (TCGA)—2136 genes (left) and 44 genes (right)

Hallmark-tsne^a^	BasL^b^		ErbB2^c^		LumA^d^		LumB^e^		NormL^f^		notSure^g^	
1	24	21	27	27	466	464	125	125	6	6	82	82
2	105	104	6	6	12	12	3	3	6	6	18	18
3	12	12	5	5	54	56	15	15	1	1	18	18

^a^Hallmark-tsne: clusters based on the hallmark-tsne subtypes

^b^BasL: Basal-like

^c^ErbB2: ErbB2/HER2-enriched

^d^LumA: Luminal A

^e^LumB: Luminal B

^f^NormL: Normal-like

^g^notSure: types are uncertainly

**Fig. 5:**
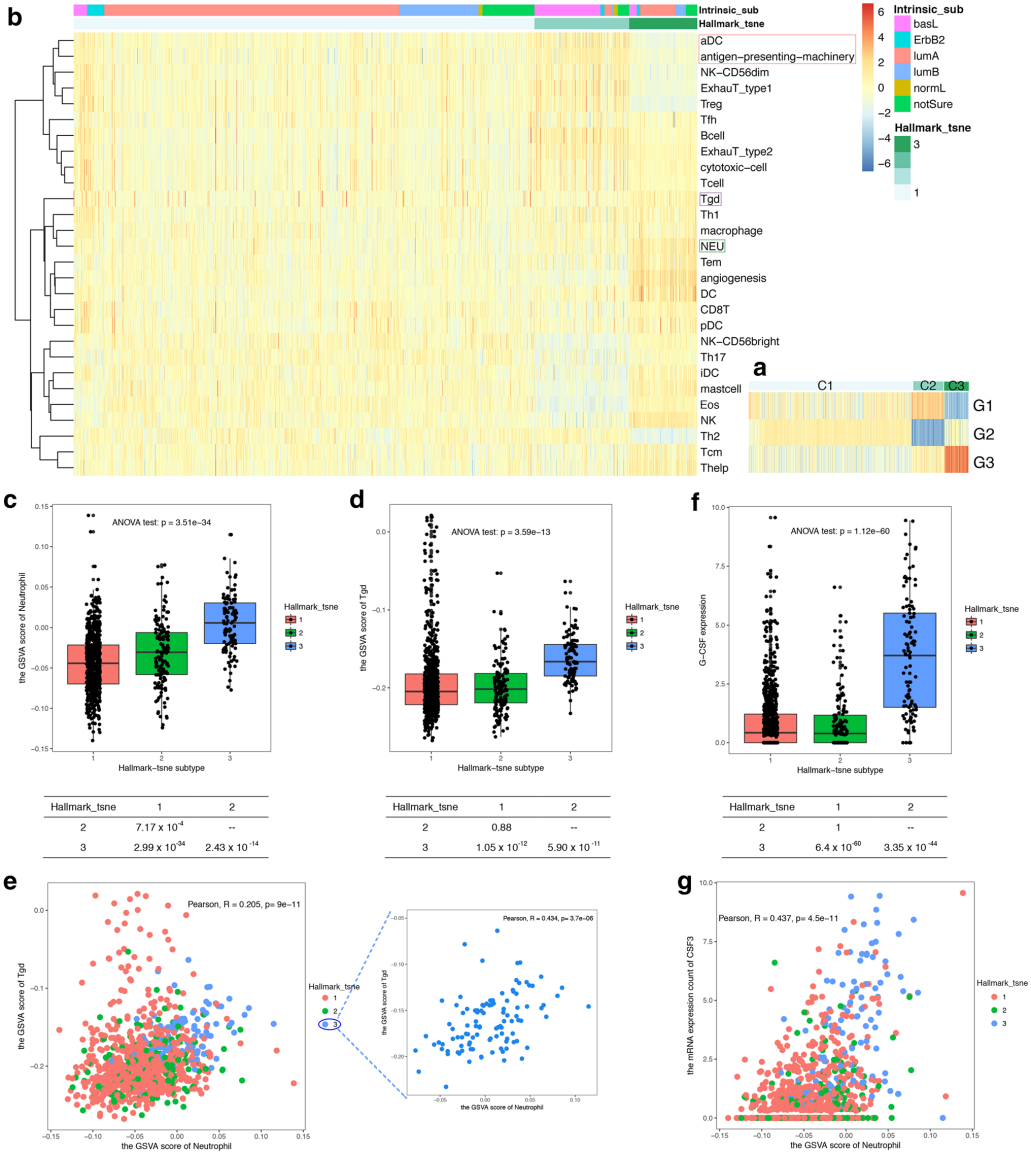
Association of infiltrating immune cell patterns with features of the Mickey-like clusters. **a** The expression status of the three comprehensive gene groups with 44 P-DEGs in the Mickey-like clusters. The vertical axis shows the three gene sets derived from the 44 P-DEGs, and the horizontal axis shows the samples ordered by hallmark-tsne subtype. The heatmap was simplified in Fig. 4b. Each cell of the heatmap represents a GSVA enrichment score based on the G1, G2 and G3 gene sets, and the score decreases from red to blue. (The legend is same as that for Fig. 4b.) **b** The distribution of tumor-infiltrating immune cells in the Mickey-like clusters. The y-axis displays the immune cell types ordered with Ward linkage in a hierarchical cluster. The x-axis depicts the samples in Mickey-like clusters and the intrinsic gene subtype order. Moreover, the GSVA scores were centered and scaled in the row direction in the heatmap. aDC: activated dendritic cell; NK-CD56^dim^: natural killer cell-CD56^dim^; ExhauT-type1: exhausted T cell-type 1; ExhauT-type2: exhausted T cell-type 2; Tgd: T gamma delta cell; NEU: neutrophil cell; Tem: T effector memory cell; DC: dendritic cell; pDC: plasmacytoid DC; iDC: immature dendritic cell; Eos: eosinophil granulocyte; Tcm: T central memory cell. **c** and **d** Distribution of the infiltrated neutrophils (**c**) and Tgd (**d**) estimated by GSVA among the hallmark-tsne subtype (ANOVA test with pairwise comparison adjusted with the Bonferroni correction). **e** Relationship of infiltrated neutrophils and Tgd cells (Pearson’s correlation). **f** Expression of G-CSF genes in the hallmark-tsne subtype (ANOVA test with pairwise comparison adjusted with the Bonferroni correction). **g** Relationship of infiltrated neutrophils and G-CSF expression counts (Pearson’s correlation)

In addition, to further investigate the features of the hallmark-tsne subtypes, we observed the mRNA expression of 44 predominate P-DEGs among the Mickey-like clusters (Fig. 4b). The 44 P-DEGs were divided into three groups based on a hierarchical cluster analysis. The genes of group 1 (G1) were focused on the HALLMARK_G2M_CHECKPOINT term and are related to proliferation (Fig. 4d). The genes of group 2 (G2) were centered on the HALLMARK_ESTROGEN_RESPONSE_LATE term, which is hormone-related and highly expressed in the luminal subtype (Fig. 4e). The third group (G3) concentrated mainly on HALLMARK_MYOGENESIS, which involves cell motility (Fig. 4f). Consistent with conventional views, the basal-like dominant C2 breast cancers possessed strong proliferative ability, moderate motility potential and a low expression of luminal-associated markers. In contrast, the high-risk C3 cluster contradicted the traditionally held view that patients with luminal subtypes exhibit a better prognosis, display extremely low proliferation status, have moderate hormone-related gene expression and exhibit highly increased motility (Fig. 5a).

### Immune infiltrating pattern of the Mickey-like clusters

To further elucidate why there is a motility-increased high-risk luminal A subtype, we evaluated the tumor microenvironment, specifically the composition of the tumor-infiltrating immune cells (Fig. 5b). The C3 tumors manifested an infiltrating immune cell pattern distinct from that of the other two clusters: (1) more T gamma delta cells (Tgd) and neutrophils aggregated in the microenvironment than in the other two subtypes (Fig. 5c, d) [24] and exhibited a positive correlation with one another in the C3 subtype (Pearson’s correlation analysis, *R* = 0.434, *p* = 3.6 × 10^−6^, Fig. 5e); (2) activated angiogenesis; (3) accumulated dendritic cells (DCs) but predominately immature DCs (iDCs) and a few activated DCs (aDCs); (4) defects of the antigen-presenting machinery (APM), which encodes MHC-I subunits and proteins essential for processing antigens and matching them onto MHC-I [13]; and (5) although many more natural killer (NK) cells than in the other two clusters, few NK-CD56^dim^ cells, which were equipped with the perforin and granular enzyme to kill tumor cells, were recruited instead of more NK-CD56^bright^ cells; and (6) impaired balance between Th1 and Th2 cells (Fig. S4), with more infiltrating Th1 cells compared with C1 (Fig. S4a) and fewer infiltrating Th2 cells compared with C1 and C2 (Fig. S4b), but still with a Th2 polarization status. All the broken or abnormal interactions between tumor cells and infiltrating immune cells generated a suitable microenvironment for the survival of C3 tumors.

For the basal-like subtypes, a small fraction classified as C1 was infiltrated by many CD8-positive T cells (CD8T), whereas the C2 tumors were CD8 deficient. Similarly, basal-like cancers were heterogeneous in terms of prognosis, and CD8T infiltration was an independent favorable prognostic indicator as previously reported [25, 26].

### NET formation increased cancer cell motility

**Fig. 6:**
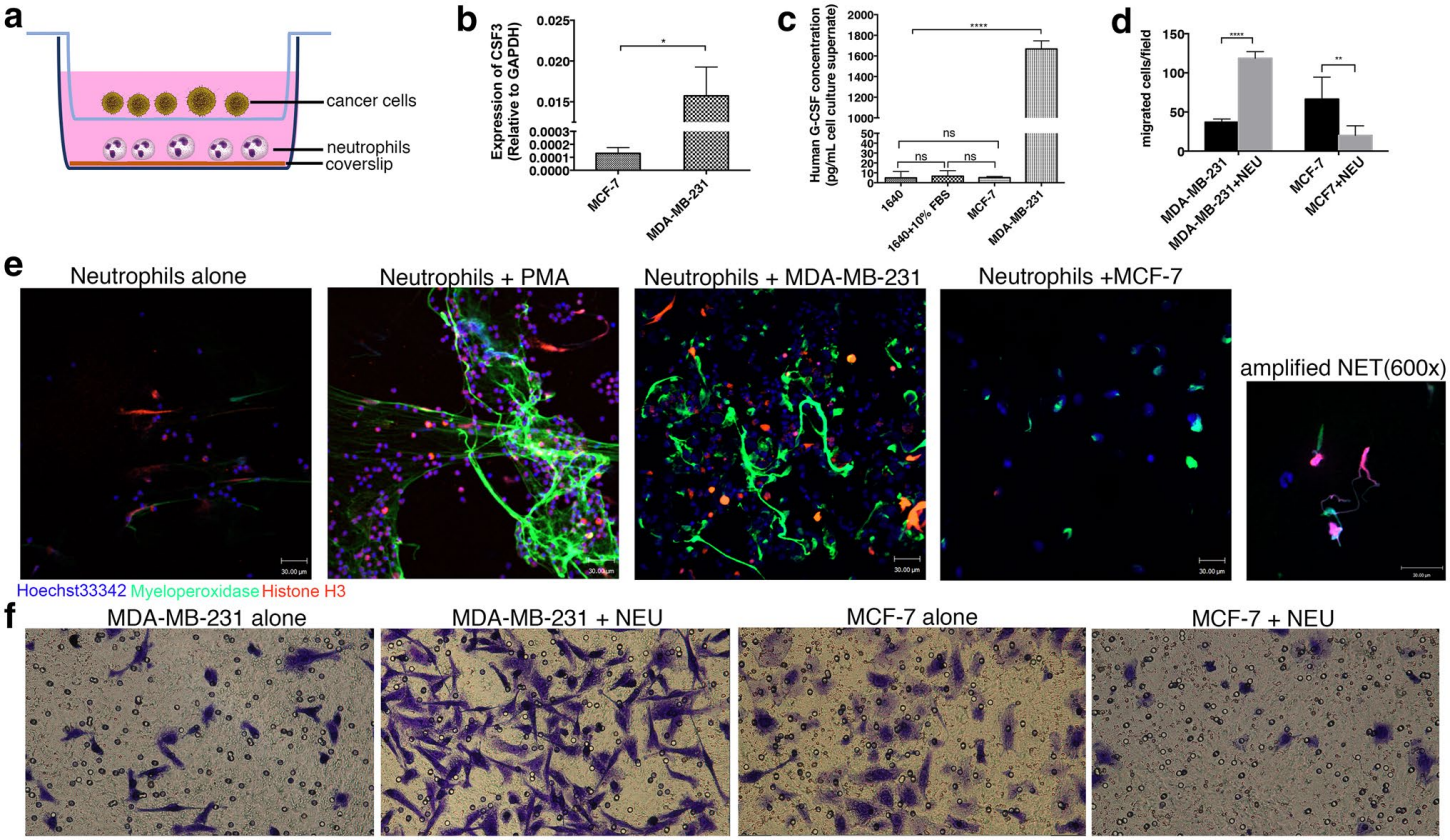
NETs formed by G-CSF high-expression MDA-MB-231 cells promote cancer cell migration. **a** Schematic of the migration/NET formation transwell assay. Fresh neutrophils were seeded on the coverslips layered in the lower chamber, and cancer cells were placed in the upper chamber. **b** and **c** Production counts of G-CSF in MDA-MB-231 and MCF-7 cells detected by qRT-PCR (mean ± SEM; t test) and ELISA (mean ± SEM; ANOVA) assays. **d** and **f** Neutrophils increased the motility of MDA-MB-231 cells but not MCF-7 cells (mean ± SEM; two-way ANOVA with multiple comparisons). **e** Unstimulated neutrophils and neutrophils primed by PMA, MDA-MB-231 cells and MCF-7 cells showed different statuses (immunostaining for myeloperoxidase and histone H3). PMA and MDA-MB-231 cells prime neutrophils into NETs. Significant differences are indicated with *, whereas nonsignificant differences are indicated with ns. *p < 0.05; **p < 0.01; ***p < 0.001; ****p < 0.0001

After integrating the expression of 44 P-DEGs and the immune cell infiltration pattern, we noted that the C3 tumors were enriched in neutrophils (Fig. 5c) and highly expressed the G3 gene (Fig. 5a). Additionally, we observed that G-CSF, a cell factor that can prime neutrophils to form NETs [27, 28], was highly expressed in C3 (Fig. 5f) tumors and showed a positive correlation with the recruitment status of neutrophils (Pearson’s correlation analysis, *R* = 0.437, *p* = 4.5 × 10^−11^, Fig. 5g). Therefore, we hypothesized that aggregated neutrophils, which can form NETs primed by G-CSF, contributed to the increased motility of C3 [14], thus promoting the dissemination of tumor cells and aggravating the illness. To assess the relationship between NET formation and tumor cell motility, we performed a coculture transwell migration assay (the details are provided in the “Materials and Methods” section, Fig. 6a) with two cell lines: MDA-MB-231 cells, which show a high production of G-CSF, and MCF-7, which rarely secretes G-CSF (Fig. 6b, c). The purity of the neutrophils used in the assay was approximately 90%, as evaluated by flow cytometry and multilobular nucleus counting (Fig. S5). The neutrophils induced NET formation after a 3-h stimulation with recombinant human G-CSF (Fig. S6a and b). In this assay, neutrophils cocultured with MDA-MB-231 cells formed extensive NETs, whereas neutrophils cocultured with MCF-7 cells formed few NETs (Figs. 6e, S6c, and S7b). Moreover, NET formation increased MDA-MB-231 cell migration; however, nonactivated neutrophils had the opposite effect on MCF-7 cell migration (Fig. 6d, f). In contrast, NETs stimulated by the exogenous human G-CSF increased the mobility of the luminal cells MCF-7 (Fig. S6d), whereas the migration ability of MDA-MB-231 cells cocultured with neutrophils was reduced when the G-CSF produced by MDA-MB-231 was neutralized (Fig. S7a and d). In parallel, NET formation was also reduced (Fig. S7b).

## Discussion

By combining the hallmark-GSVA enrichment scores and t-SNE algorithm, we discovered a high-risk motility-increased luminal A dominant breast cancer type with neutrophil aggregation (C3) in TCGA data. Concordant with traditional views, breast cancer is not a single disease [1], and the existing intrinsic and Claudin-low (CL) subtypes are not sufficient to cover all subtypes. In attempting to elucidate the reasons for differing prognoses, we delineated hallmark-tsne subtypes, particularly C3, including (1) molecular characterization with prognosis-associated hallmark terms and 44 P-DEGs and the (2) tumor-infiltrating immune cell pattern.

Cancer stem cells (CSCs) are causally regarded as the seeds of tumor relapse and metastasis that activate the same signaling pathways that are expressed in normal stem cells, such as hedgehog, Wnt and notch [29]. CSCs have been identified in brain, breast and pancreatic cancers [30–32]. In breast cancer, the activated hedgehog pathway increases the numbers of mammosphere-initiating cells and regulates the self-renewal of tumor-initiating cells [31]. From the HGS profiles, the C3 tumors showed a CSC phenotype with highly expressed hedgehog signaling. Previously, it has been shown that one distinguished phenotype of C3 tumors is dependent on fatty acid metabolism and is paramount to maintain the CSC phenotype in breast mesenchymal CSCs [33]. In addition, the expression pattern of 44 P-DEGs reveals that the C3 tumors are endowed with increased motility abilities. In terms of the above molecular features, the C3 subtype displayed increased motility and a cancer stem-like phenotype in the same manner as breast circulating tumor cells [34].

Alternatively, the peculiar pattern of tumor-infiltrating immune cells in C3 tumors is critical to their worse prognosis. First, Tgd cells are concordant with neutrophil accumulation. The mechanism of this seeming coincidence is that tumor cells elicit IL17 expression from Tgd cells, resulting in a systemic inflammation cascade (expansion and polarization of neutrophils). Herein, the IL17-producing Tgd cells and neutrophils together promote breast cancer metastasis [24]. Alternatively, the G-CSF production in C3 tumors recruits and primes neutrophils to form metastases supporting NETs. The metastasis-promoting cascade is activated, and the tumor-attacking system is broken. The defects in the APM interrupt the communication between tumor antigens and cytotoxic cells, resulting in a lack of tumor-specific attacking cells.

In addition, there is another interesting finding from the pattern of tumor-infiltrating immune cells. In general, cancer patients exhibit an imbalanced ratio of Th1 and Th2 cells, shifting from the anti-tumor Th1 cells that are responsible for tumor immune surveillance to the pro-tumor Th2 cells that are associated with tumor immune evasion [35]. The same phenomenon was observed in the TCGA data. However, the C2 and C3 subtypes, which are associated with a worse prognosis, were, in contrast to the traditional perspective, infiltrated with more Th1 cells than was the C1 subtype. Although most of the evidence supports that patients with more infiltrating Th1 cells exhibit a good prognosis, there is also research that indicates that patients with more infiltrating Th1 cells suffer a poorer prognosis compared with those with fewer Th1 cells, as evaluated by flow cytometry. The data from that study indicated that the IFN-γ secreted by Th1 cells could enhance the PD-L1 signal in macrophages and tumor cells [36]. Even though the Th1 and Th2 status was estimated from the bulk RNA sequencing indirectly, the information suggests that the roles of T helper cells in the tumor microenvironment are more complicated and merit further investigation.

To our knowledge, C3 is a minor novel subtype. Although C3 is similar to a Claudin-low subtype in some aspects, such as incidence (approximately 10%) [37] and cancer stem cell-like features, these subtypes differ from one another in the following aspects: (1) hormone receptor status—C3 is dominant in luminal A subtypes, whereas triple-negative (TN) tumors account for more than half of CL tumors [17]; and (2) prognosis - CL behaves more poorly than the luminal subtype but better than the basal-like subtype from METABRIC datasets (Fig. S8); C3 has worse prognosis even compared with the basal-like dominant C2.

The t-distributed stochastic neighbor embedding (t-SNE) method [20] is another highlight of this study. This method is a nonlinear dimensionality reduction technique that specializes in simplifying high-dimensional data into a low-dimensional space, typically the 2D plane. The t-SNE algorithm has been applied in mass cytometry [38] and in single-cell RNA sequencing [19], but not in bulk RNA sequencing data until now. This study constitutes the first attempt to explore potential subtypes in breast cancer with the nonlinear cluster method of t-SNE.

In summary, we identified a high-risk breast cancer subtype that displayed increased motility abilities, decreased proliferation capacity, and other CSC-like features, a high expression of hormone/luminal-related genes and immune dysfunction (neutrophil aggregation and APM defects). Thus, the biological processes and immune heterogeneity of breast cancer must be understood to facilitate the improvement of clinical treatments. For example, characterizing the minor C3 subtype has pressing clinical implications with regard to specific treatments, such as deoxyribonuclease I (DNAase I) to digest NETs or the use chimeric antigen receptor T-cell immunotherapy (CAR-T) to remedy the antigen-presenting dysfunction.

## Electronic supplementary material

Below is the link to the electronic supplementary material.

Supplementary material 1 (TIF 728 KB)

Supplementary material 2 Fisher’s exact test was used to evaluate the proportion of ER/PR/HER2-positive samples in hallmark-tsne subtypes. (TIF 1087 KB)

Supplementary material 3 (TIF 157 KB)

Supplementary material 4 Infiltrating Th1 (a) and Th2 (b) estimated by GSVA among the hallmark-tsne subtype (ANOVA test with pairwise comparison adjusted with the Bonferroni correction). The ratio of Th2 to Th1 populations was calculated with the 2^n transformed GSVA scores (c). (TIF 549 KB)

Supplementary material 5 (a) The neutrophil purity was evaluated by flow cytometry and exhibited forward and side scatter (FSC and SSC); as demonstrated by staining, the neutrophils expressed certain markers (CD15 and CD16) and did not express other markers (CD49d, which is expressed on other PMN and monocytes). A total of 50000 events were acquired, and the percentage of CD49d- events is provided under gate Q2. (b) To count the multilobular nuclei, the isolated cells were stained with Hoechst 33342, and the percentage of the cells with multilobular nuclei from five independent experiments are shown on the top right corner. (TIF 1599 KB)

Supplementary material 6 (a) The results of ELISA of the MPO:DNA complex showed that neutrophils can form NETs when stimulated by recombinant human G-CSF (mean ± SEM; n = 3, t-test). (b) Representative images of the untreated neutrophils and the NETs induced by G-CSF. (c) MCF-7 cells stimulated fewer NETs (mean ± SEM; n = 7, t-test) than MCF-7 cells supplemented with exogenous human G-CSF (6 ng/mL; mean ± SEM; n = 3, t-test). (d) The NETs induced by the exogenous human G-CSF increased the migration ability of MCF-7 cells (mean ± SEM; n = 4, t-test) compared with untreated MCF-7 cells (mean ± SEM; n = 6, t-test). Representative images of the migrated MCF-7 cells and the formed NETs in the MCF-7, neutrophil and exogenous human G-CSF assay. (TIF 12165 KB)

Supplementary material 7 The migration ability of MDA-MB-231 cell lines (a and d) and NET formation (b and c) were reduced by neutralizing G-CSF secreted by the cell line (mean ± SEM; n ≥ 3, t-test). (TIF 9491 KB)

Supplementary material 8 Claudin-low breast cancer from METABRIC datasets exhibits a worse prognosis than the luminal subtype but better survival than the basal-like subtype. (TIF 524 KB)

Supplementary material 9 The gene list of exhausted T cells (XLS 27 KB)

Supplementary material 10 (XLSX 119 KB)
