# Supplementary figures and images for: A high-risk luminal A dominant breast cancer subtype with increased mobility
Source: Breast Cancer Res Treat. 2019 Feb 19;175(2):459–72. doi: 10.1007/s10549-019-05135-w (PMC6533414; doi:10.1007/s10549-019-05135-w)

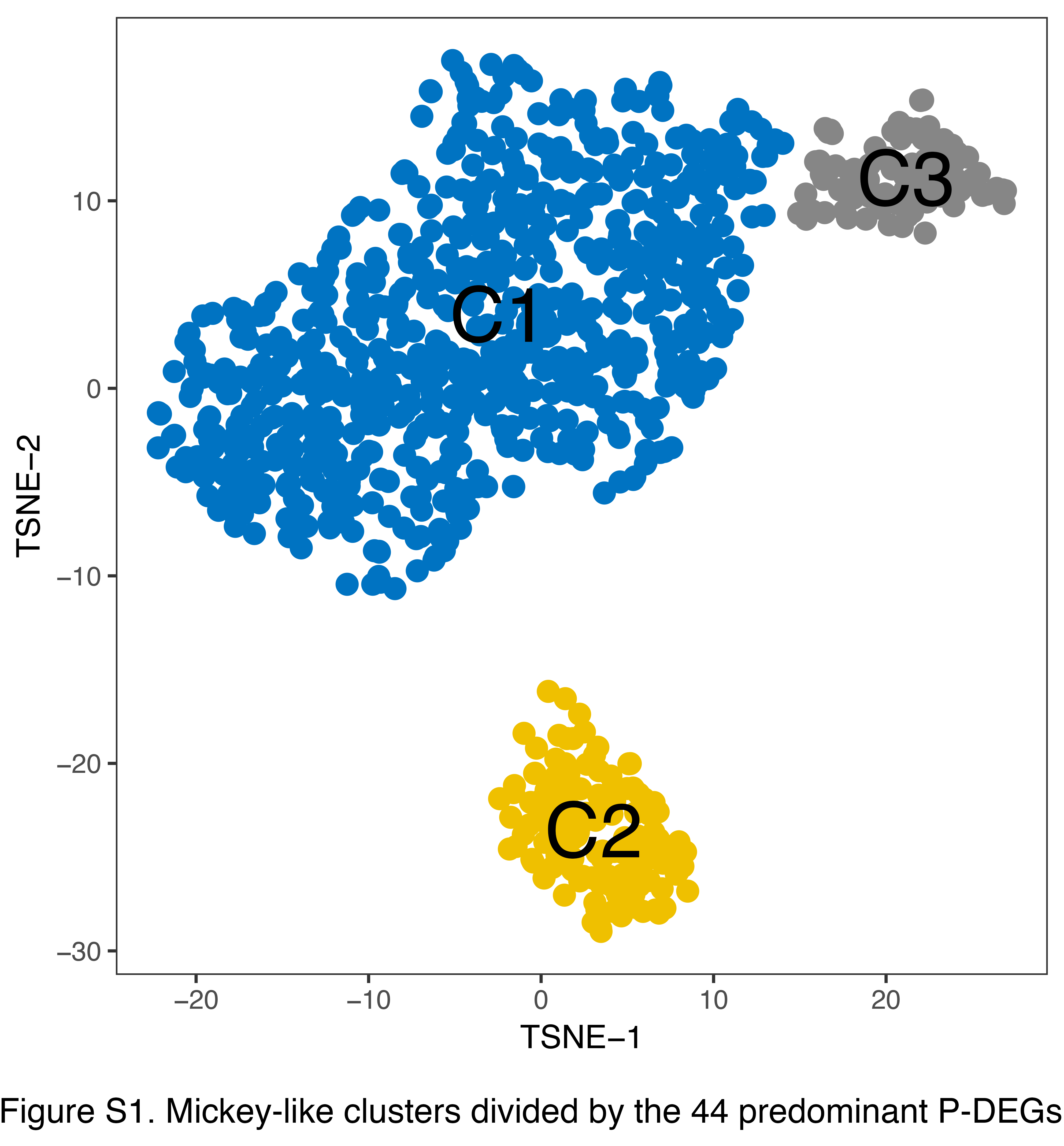

Supplement: Supplementary file 1 — Supplementary material 1 (TIF 728 KB) [file 10549_2019_5135_MOESM1_ESM.tif]

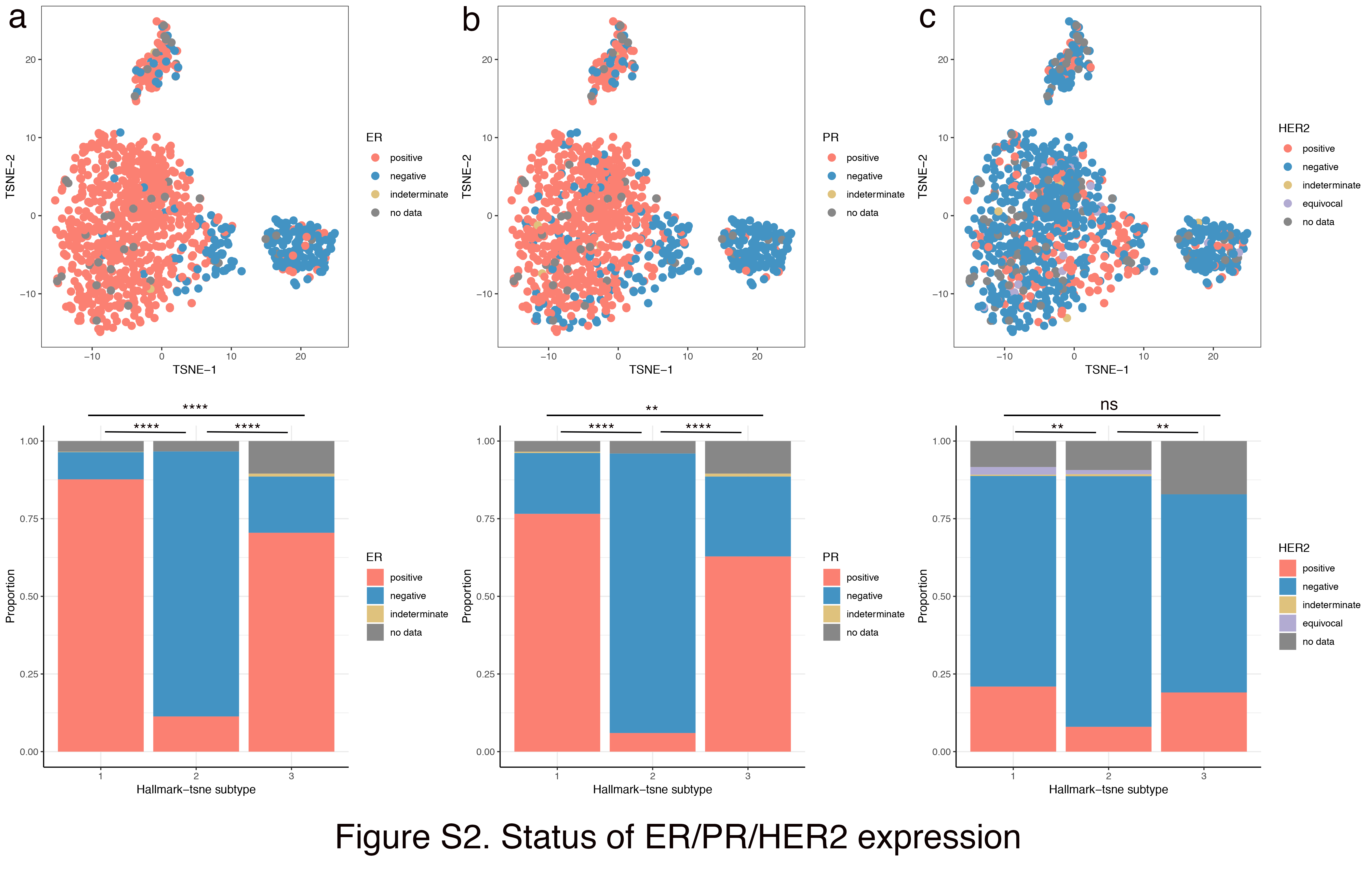

Supplement: Supplementary file 2 — Supplementary material 2 Fisher’s exact test was used to evaluate the proportion of ER/PR/HER2-positive samples in hallmark-tsne subtypes. (TIF 1087 KB) [file 10549_2019_5135_MOESM2_ESM.tif]

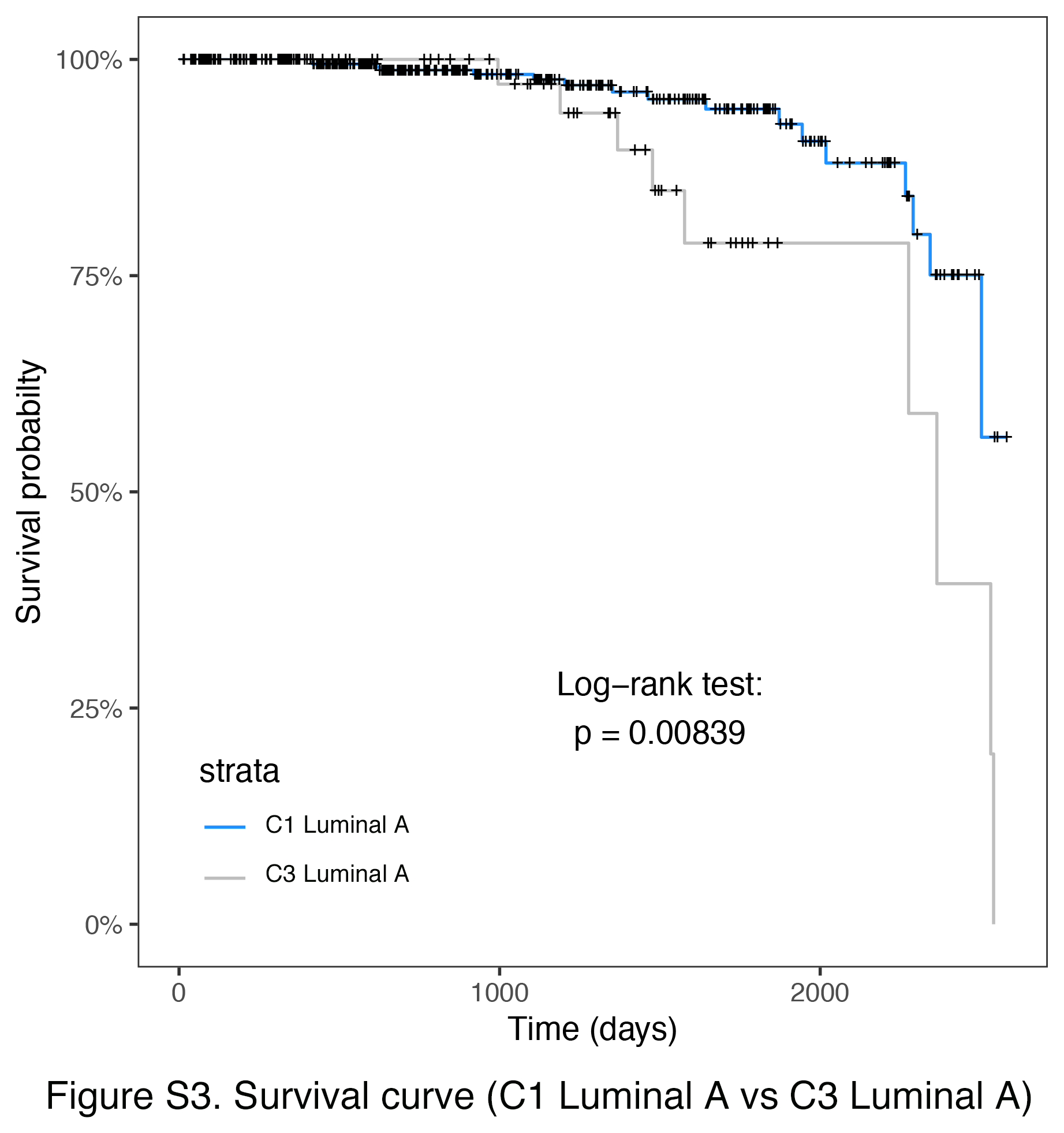

Supplement: Supplementary file 3 — Supplementary material 3 (TIF 157 KB) [file 10549_2019_5135_MOESM3_ESM.tif]

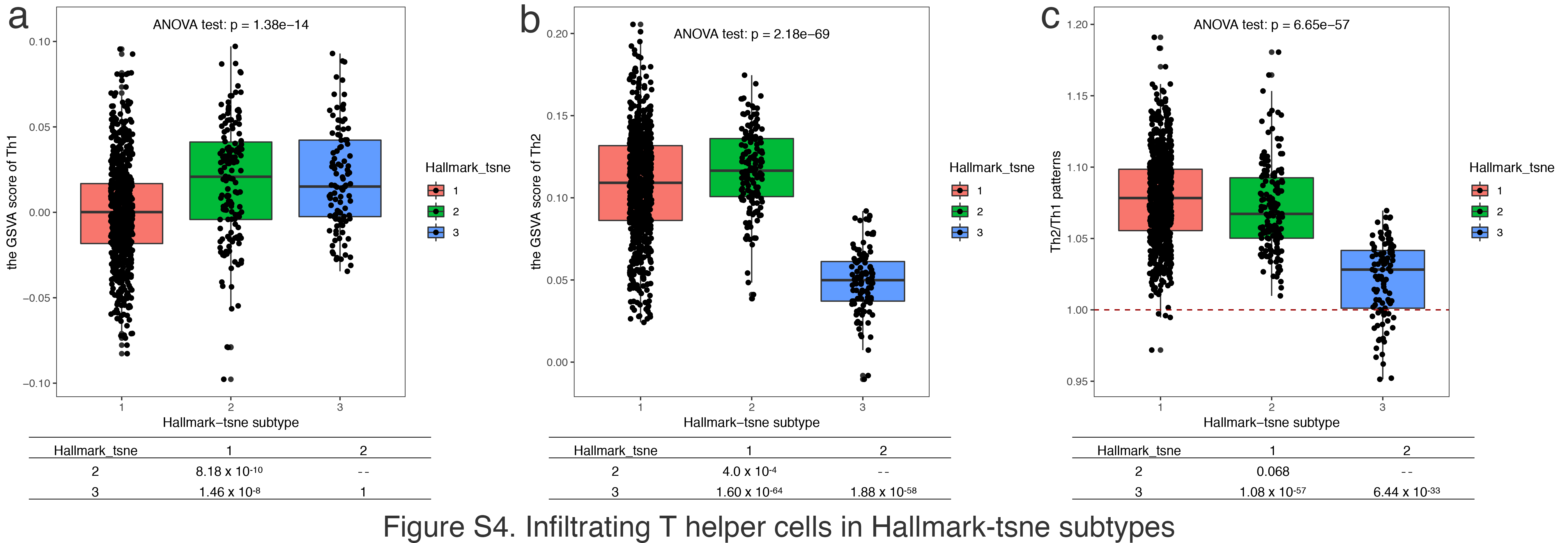

Supplement: Supplementary file 4 — Supplementary material 4 Infiltrating Th1 (a) and Th2 (b) estimated by GSVA among the hallmark-tsne subtype (ANOVA test with pairwise comparison adjusted with the Bonferroni correction). The ratio of Th2 to Th1 populations was calculated with the 2^n transformed GSVA scores (c). (TIF 549 KB) [file 10549_2019_5135_MOESM4_ESM.tif]

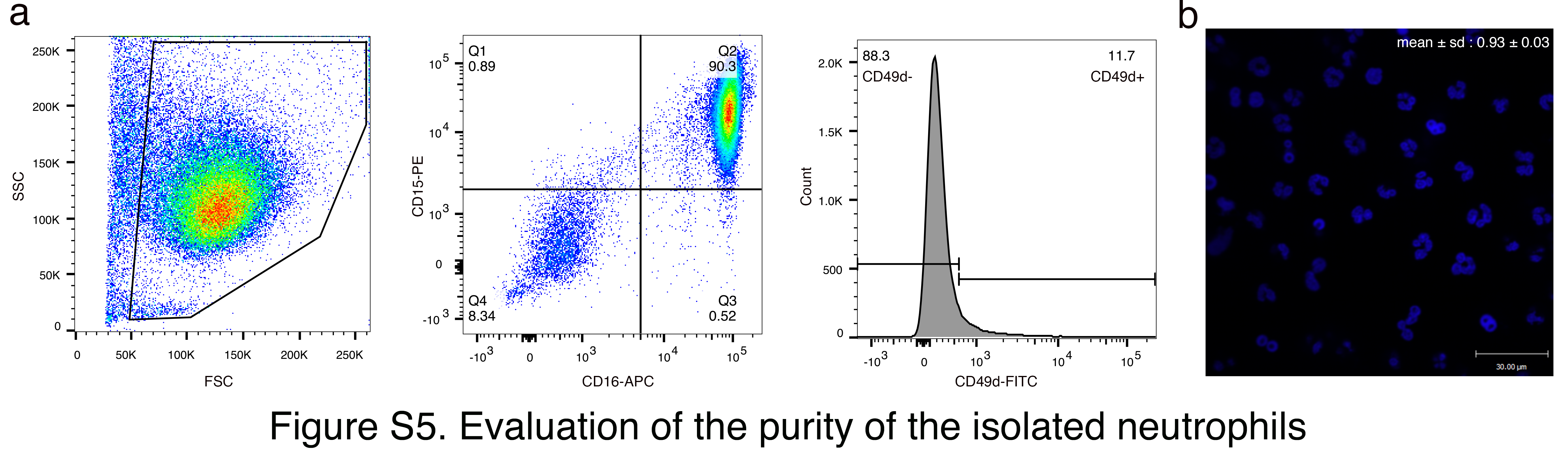

Supplement: Supplementary file 5 — Supplementary material 5 (a) The neutrophil purity was evaluated by flow cytometry and exhibited forward and side scatter (FSC and SSC); as demonstrated by staining, the neutrophils expressed certain markers (CD15 and CD16) and did not express other markers (CD49d, which is expressed on other PMN and monocytes). A total of 50000 events were acquired, and the percentage of CD49d- events is provided under gate Q2. (b) To count the multilobular nuclei, the isolated cells were stained with Hoechst 33342, and the percentage of the cells with multilobular nuclei from five independent experiments are shown on the top right corner. (TIF 1599 KB) [file 10549_2019_5135_MOESM5_ESM.tif]

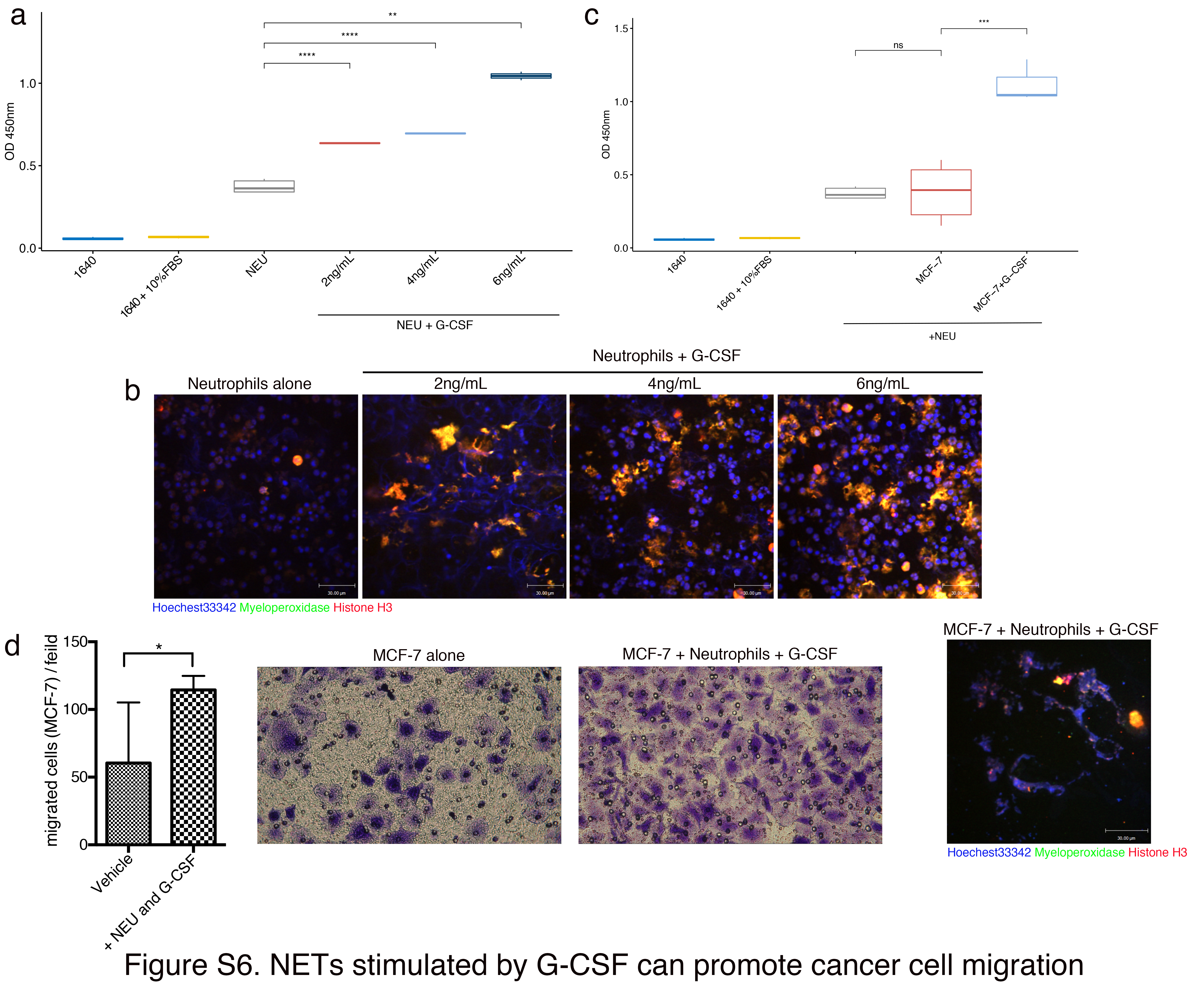

Supplement: Supplementary file 6 — Supplementary material 6 (a) The results of ELISA of the MPO:DNA complex showed that neutrophils can form NETs when stimulated by recombinant human G-CSF (mean ± SEM; n = 3, t-test). (b) Representative images of the untreated neutrophils and the NETs induced by G-CSF. (c) MCF-7 cells stimulated fewer NETs (mean ± SEM; n = 7, t-test) than MCF-7 cells supplemented with exogenous human G-CSF (6 ng/mL; mean ± SEM; n = 3, t-test). (d) The NETs induced by the exogenous human G-CSF increased the migration ability of MCF-7 cells (mean ± SEM; n = 4, t-test) compared with untreated MCF-7 cells (mean ± SEM; n = 6, t-test). Representative images of the migrated MCF-7 cells and the formed NETs in the MCF-7, neutrophil and exogenous human G-CSF assay. (TIF 12165 KB) [file 10549_2019_5135_MOESM6_ESM.tif]

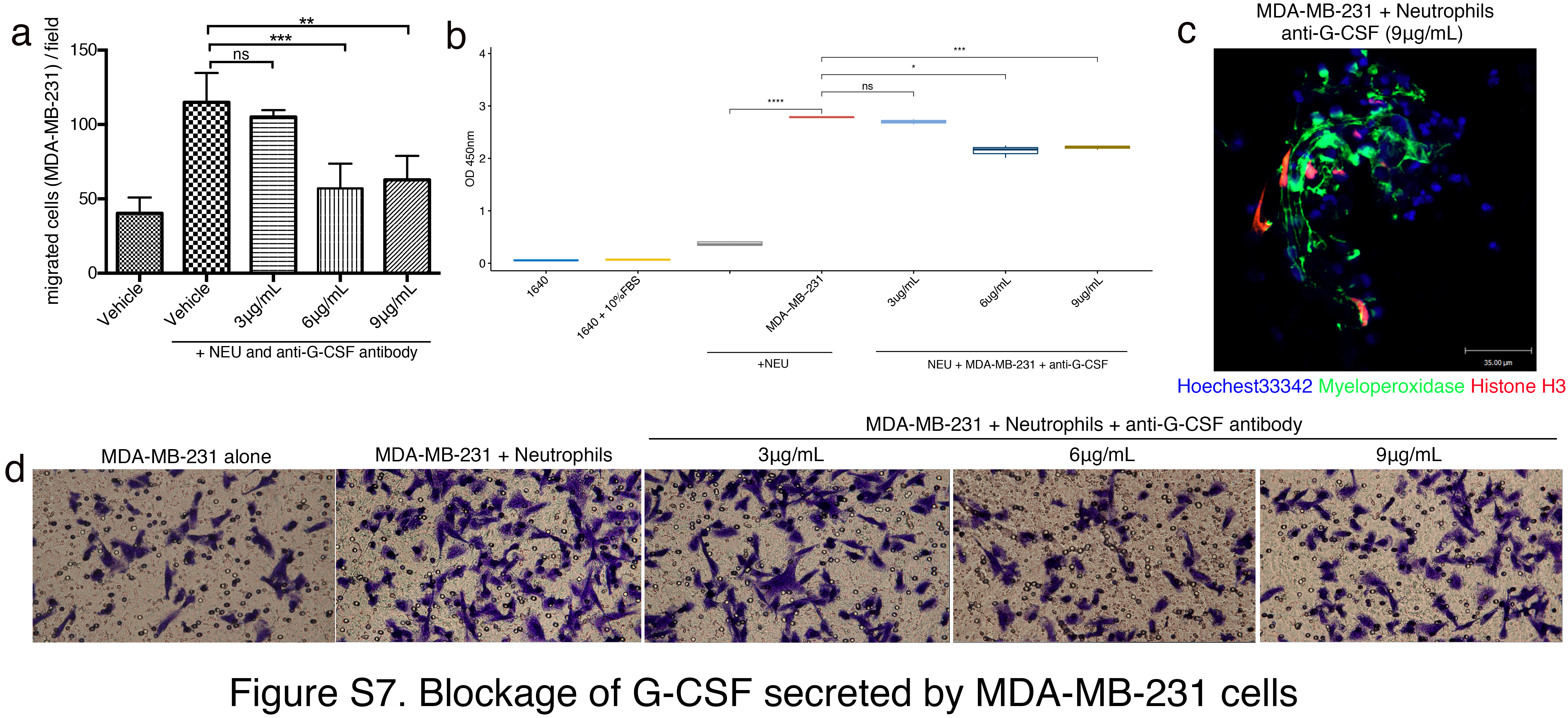

Supplement: Supplementary file 7 — Supplementary material 7 The migration ability of MDA-MB-231 cell lines (a and d) and NET formation (b and c) were reduced by neutralizing G-CSF secreted by the cell line (mean ± SEM; n ≥ 3, t-test). (TIF 9491 KB) [file 10549_2019_5135_MOESM7_ESM.tif]

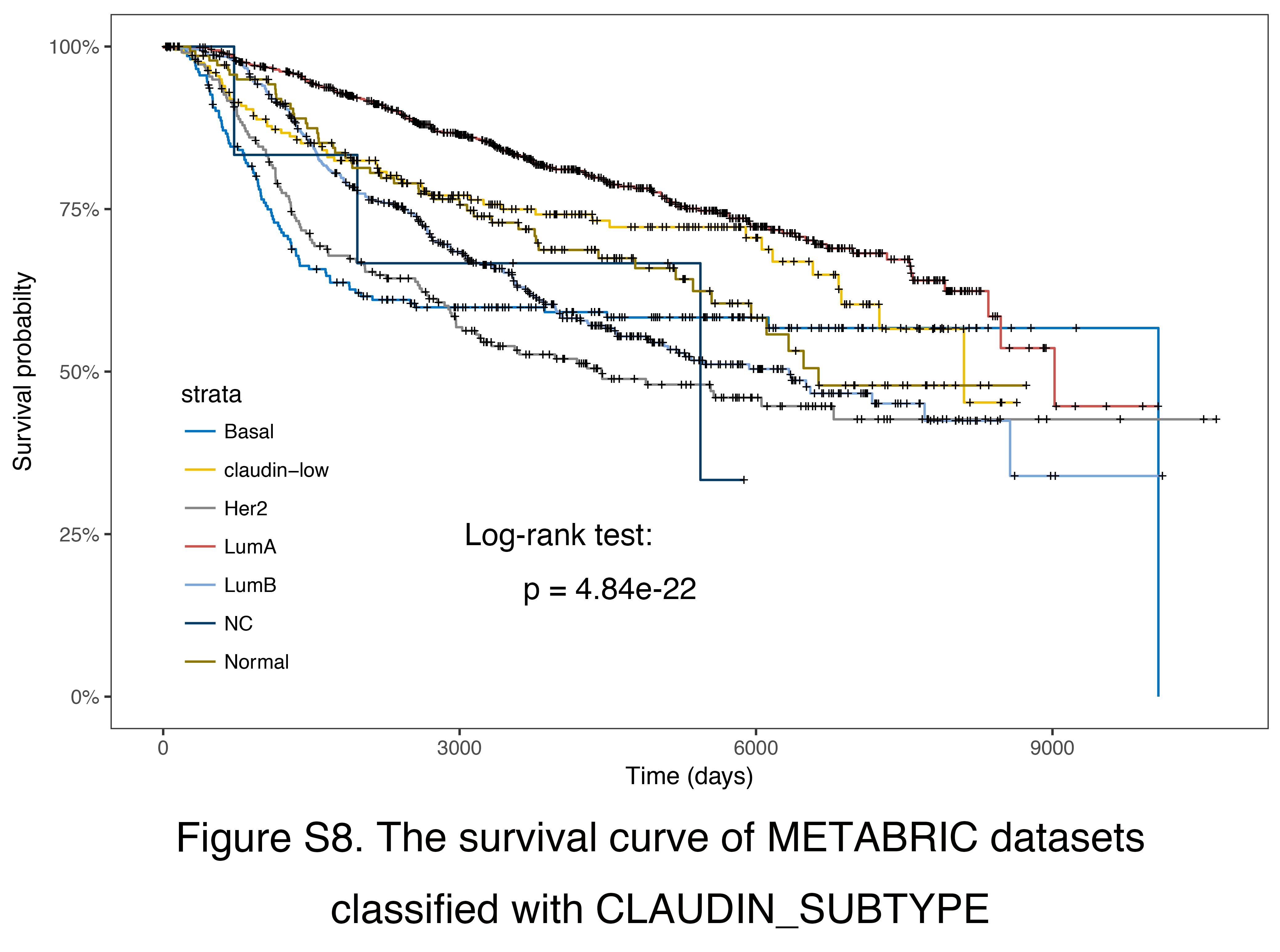

Supplement: Supplementary file 8 — Supplementary material 8 Claudin-low breast cancer from METABRIC datasets exhibits a worse prognosis than the luminal subtype but better survival than the basal-like subtype. (TIF 524 KB) [file 10549_2019_5135_MOESM8_ESM.tif]
